# Fluctuating and Geographically Specific Selection Characterize Rapid Evolution of the Human *KIR* Region

**DOI:** 10.3389/fimmu.2019.00989

**Published:** 2019-05-17

**Authors:** Danillo G. Augusto, Paul J. Norman, Ravi Dandekar, Jill A. Hollenbach

**Affiliations:** ^1^Department of Neurology, University of California, San Francisco, San Francisco, CA, United States; ^2^Division of Biomedical Informatics and Personalized Medicine, Department of Immunology, University of Colorado, Denver, CO, United States

**Keywords:** killer cell immunoglobulin-like receptors, evolution, human populations, diversity, pathogens, imputation

## Abstract

The *killer-cell immunoglobulin-like receptor* (*KIR*) region comprises a fast-evolving family of genes that encode receptors for natural killer (NK) cells and have crucial role in host defense. Evolution of *KIR* was examined in the context of the human genome. Gene-content diversity and single nucleotide polymorphisms (SNP) in the *KIR* genes and flanking regions were compared to >660,000 genome-wide SNPs in over 800 individuals from 52 populations of the human genome diversity panel (HGDP). *KIR* allelic diversity was further examined using next generation sequencing in a subset of 56 individuals. We identified the SNP *rs587560* located in *KIR3DL3* as a marker of *KIR2DL2* and *KIR2DL3* and, consequently, Cen A and Cen B haplotypes. We also show that combinations of two *KIR2DL4* SNPs (*rs35656676* and *rs592645*) distinguish *KIR3DL1* from *KIR3DS1* and also define the major *KIR3DL1* high- and low-expressing alleles lineages. Comparing the diversity of the SNPs within the *KIR* region to remainder of the genome, we observed a high diversity for the centromeric *KIR* region consistent with balancing selection (*p* < 0.01); in contrast, centromeric *KIR* diversity is significantly reduced in East Asian populations (*p* < 0.01), indicating purifying selection. By analyzing SNP haplotypes in a region spanning ~500 kb that includes the *KIR* cluster, we observed evidence of strong positive selection in Africa for high-expressing *KIR3DL1* alleles, favored over the low-expressing alleles (*p* < 0.01). In sharp contrast, the strong positive selection (*p* < 0.01) that we also observed in the telomeric *KIR* region in Oceanic populations tracked with a high frequency of *KIR3DS1*. In addition, we demonstrated that worldwide frequency of high-expression *KIR3DL1* alleles was correlated with virus with virus (r = 0.64, *p* < 10^−6^) and protozoa (r = 0.69, *p* < 10^−6^) loads, which points to selection globally on *KIR3DL1* high-expressing alleles attributable to pathogen exposure.

## Introduction

Due to their pivotal role in the immune response, much attention has been given recently to variation in the highly polymorphic killer cell immunoglobulin-like receptors (KIR), expressed on the surface of natural killer (NK) (1) cells and a subset of T cells (2). The *KIR* gene family co-evolves with the genes that encode the human leukocyte antigen (HLA) class I molecules, the ligands for most KIR molecules (3–5). KIR transduce inhibitory and/or activating signals that regulate NK cell activation, and specific *KIR* and *HLA* combinations have been associated with numerous diseases, including autoimmunity, cancer and infection (6–10). In addition, KIR-HLA combinations also impact reproduction and placentation (11–14).

The unusual structural polymorphism of the *KIR* region, yielding variable presence or absence for most of the *KIR* genes (and thus numerous observed gene-content haplotypes) (15) combined with pronounced allelic variation at each locus- and their demonstrated importance for human survival (16, 17) make them intriguing targets for disease association and evolutionary studies. The 15 *KIR* loci were formed by multiple duplication events and unequal crossovers (18), evolving relatively rapidly compared to other genomic regions (19–21). As a consequence, *KIR* genes share substantial sequence similarity with one another, which together with their structural polymorphism, impose technical barriers to their study, particularly at allelic level (22).

*KIR* gene-content haplotypes are generally described as belonging to two groups, *A* and *B* (23, 24), with the *A* haplotype being relatively conserved in terms of gene-content configuration and represented mostly by inhibitory genes; in contrast, the B group has significant variation in haplotypes that include different combinations of inhibitory and activating *KIR*. Although a large number of *KIR* haplotypes have been reported, the most common haplotypes are formed by combinations of four centromeric (*CenA, CenB1, CenB2*, and *CenB3*) and two telomeric (*TelA* and *TelB*) configurations of *KIR* genes (25, 26). The framework genes are those that are present in almost all haplotypes and flank the centromeric and telomeric regions of the *KIR* haplotypes. Flanking the centromeric segment of the region are *KIR3DL3* and *KIR3DP1*, while *KIR2DL4* and *KIR3DL2* flank the telomeric portion.

Here, we analyzed publicly available data for over 660,000 single nucleotide polymorphisms (SNPs) in the context of *KIR* diversity in 52 populations from the well-established panel of samples from the Human Genome Diversity Project—Centre d'Etude du Polymorphisme Humain (HGDP-CEPH) (27). The HGDP-CEPH panel is a worldwide collection of population-based samples that have been analyzed with respect to hundreds of thousands of genetic variants, including presence and absence of all *KIR* genes (26). We observed compelling evidence of selection shaping the diversity of the *KIR* region in a population-specific manner. In particular, we found strong signals for positive selection in Africans favoring members of the *KIR3DL1* allelic lineage that is expressed at highest levels on the surface of NK cells.

## Methods

### Data Collection

We analyzed publicly available SNP and sequencing data for 817 individuals from 52 populations from the HGDP panel. The first subset of samples was genotyped by Illumina SNP microarray (San Diego, California, USA) and was comprised of 805 individuals from 50 populations (subset 1, Table 1), from which we analyzed a total of 660,918 SNPs extracted from two sources: 143,945 SNPs from the UCLA Medical Center Illumina Immunochip22 HGDP Dataset 15 (ftp://ftp.cephb.fr/hgdp_supp15/) and 516,973 from the Stanford HGDP SNP Genotyping Dataset 2 (http://www.hagsc.org/hgdp/files.html). The other subset (subset 2, Table 1) was comprised of 56 individuals that had been previously sequenced for the whole genome or whole exome (28, 29), in which we applied our custom bioinformatics pipeline (30) to determine *KIR* allelic genotyping at high-resolution. We analyzed the SNP and sequence data in the context of *KIR* gene content that was previously genotyped for the HGDP panel by analyzing the amplicons generated by polymerase chain reaction with specific sequence primers (PCR-SSP) using matrix-assisted laser desorption-ionization time-of-flight (MALDI-TOF).

**Table 1:** List of populations included in this study.

**Population**	**SNP data (subset 1)**	**High-resolution allelic genotyping (subset 2)**	**Region**
Bantu N.E.	9		Africa
Bantu S.	7		
Biaka Pygmies	22		
Mandenka	21	1	
Mbuti Pygmies	11	7	
San	6	5	
Yoruba	20	1	
Mozabite	26	6	Middle East
Bedouin	46		
Palestinian	38		
Druze	40		
Adygei	11		Europe
French	18	3	
French Basque	20		
North Italian	10	1	
Orcadian	12		
Russian	21		
Sardinian	24	1	
Tuscan	8		
Pathan	21	7	Central and South Asia
Makrani	25		
Kalash	22		
Hazara	20		
Balochi	22		
Barusho	19		
Brahui	21		
Sindhi	13		
Cambodia	0	5	
Uygur	9		
Dai	7	1	East Asia
Daur	9		
Han	42	1	
Hezhen	10		
Japanese	23	2	
Lahu	8		
Miaozu	8		
Mongola	10		
Naxi	9		
Orogen	8		
She	10		
Tu	9		
Tujia	7		
Xibo	8		
Yakut	0	7	
Yizu	10		
Papuan	15	1	Oceania
NAN Melanesian	14		
Karitiana	14	1	America
Maya	16	6	
Pima	14		
Surui	6		
Colombian	6		
Total in each subset	805	56	
Total of unique individuals	817	

### Data Analysis

PLINK version 1.07 (31) was used for all manipulation of SNP data. We only included SNPs whose genotypic distributions did not deviate from Hardy-Weinberg equilibrium (*p* > 0.01) and with minor allele frequency (MAF) > 0.01. A total of 62 SNPs were extracted from the *KIR* region (GRCh38.p12, chr19:54727369-54865755). After quality control and merging of the two platforms, 660,918 unique SNPs were available for analysis. To compare the diversity of the SNPs within the *KIR* region to the distribution of SNPs across the whole genome, we generated distributions for heterozygosity of the SNPs in the *KIR* and genome-wide regions using the “ecdf” function in the R (32) stats package, and applied the Kolmogorov–Smirnov test using the “ks.test” function, a nonparametric test that quantifies the distance between the empirical distribution functions of two datasets (33), to examine differences in the distributions (34). To examine the association of SNPs with the presence of specific variable *KIR* loci, we first visualized linkage disequilibrium (LD) between the SNPs and the *KIR* loci using Haploview (35) to identify SNPs marking loci of interest, and subsequently manually sorted and examined their associations in Microsoft Excel. Similarly, we analyzed whether specific SNPs were associated with *KIR* alleles and allelic lineages, and manually calculated LD values.

For analysis of the extended haplotype homozygosity (EHH) (36), we analyzed 402 SNPs located within a range of 237,688 bp upstream and 121,621 bp downstream the *KIR* region, totaling 497,695 bp (GRCh38.p12, chr19:54489681-54987376). We used the R package rehh (37) after phasing the SNPs using the FastPHASE package (38) to generate bifurcation and EHH plots.

To perform correlation analysis between *KIR* frequencies and pathogen load, we used previously published data quantifying pathogen load in each HGDP population (39) using the “cor” function in the R base package. Labels for populations were randomly permuted 10,000 times and the correlations recalculated to obtain an empirical distribution for the correlation coefficient to obtain *p*-values.

## Results

### Diversity of Centromeric and Telomeric *KIR* Regions Varies Between Geographic Groups

**Figure 1:**
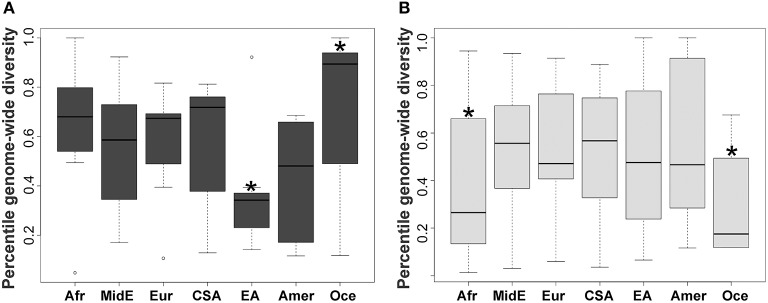
Distinct diversity patterns of centromeric and telomeric *KIR* regions. **(A)** Diversity of centromeric *KIR* SNPs relative to the distribution of genome-wide SNPs. **(B)** Diversity of telomeric *KIR* SNPs relative to the distribution of genome-wide SNPs. Horizontal bar represents mean. Asterisks mark statistical significance (*p* = 0.01) for Kolmogorov–Smirnov test. Afr, Africa; MidE, Middle East; Eur, Europe; CSA, Central and South Asia; EA, East Asia; Amer, America; Oce, Oceania.

Whereas*, KIR* diversity has been examined to-date in numerous population studies (40), without the context of genome-wide data it is difficult to disentangle whether observed diversity is a feature of population demographics, or rather indicative of a history of selection on the region. To understand how diversity within the *KIR* region compares to genome-wide diversity within populations, we examined genotypic data from over 660,000 SNPs in 805 individuals from 50 populations (Table 1) of the human genome diversity panel (HGDP). A total of 62 SNPs were located within the *KIR* gene cluster, from which 29 were in the centromeric region (*KIR3DL3* ~ *KIR3DP1*) and 33 in the telomeric region (*KIR2DL4* ~ *KIR3DL2*). Populations were grouped according to geographic region for analysis (Table 1). We compared the distribution of heterozygosity (He) of the variants within the *KIR* telomeric and centromeric regions to that for the variants across the genome in each geographic group. We observed a significantly reduced (*p* < 0.01) diversity of the centromeric *KIR* SNPs in comparison to the genome-wide diversity in East Asians, as well as increased centromeric diversity in Oceania (Figure 1A). For the telomeric region, we observed reduced diversity in Africa and Oceania compared to genome-wide (*p* < 0.01; Figure 1B).

### *KIR3DL3* Intronic Variant *rs587560* Distinguishes *CenA* and *CenB* Haplotypes and a Pair of *KIR2DL4* SNPs Defines the Major *KIR3DL1S1* Allelic Lineages

**Figure 2:**
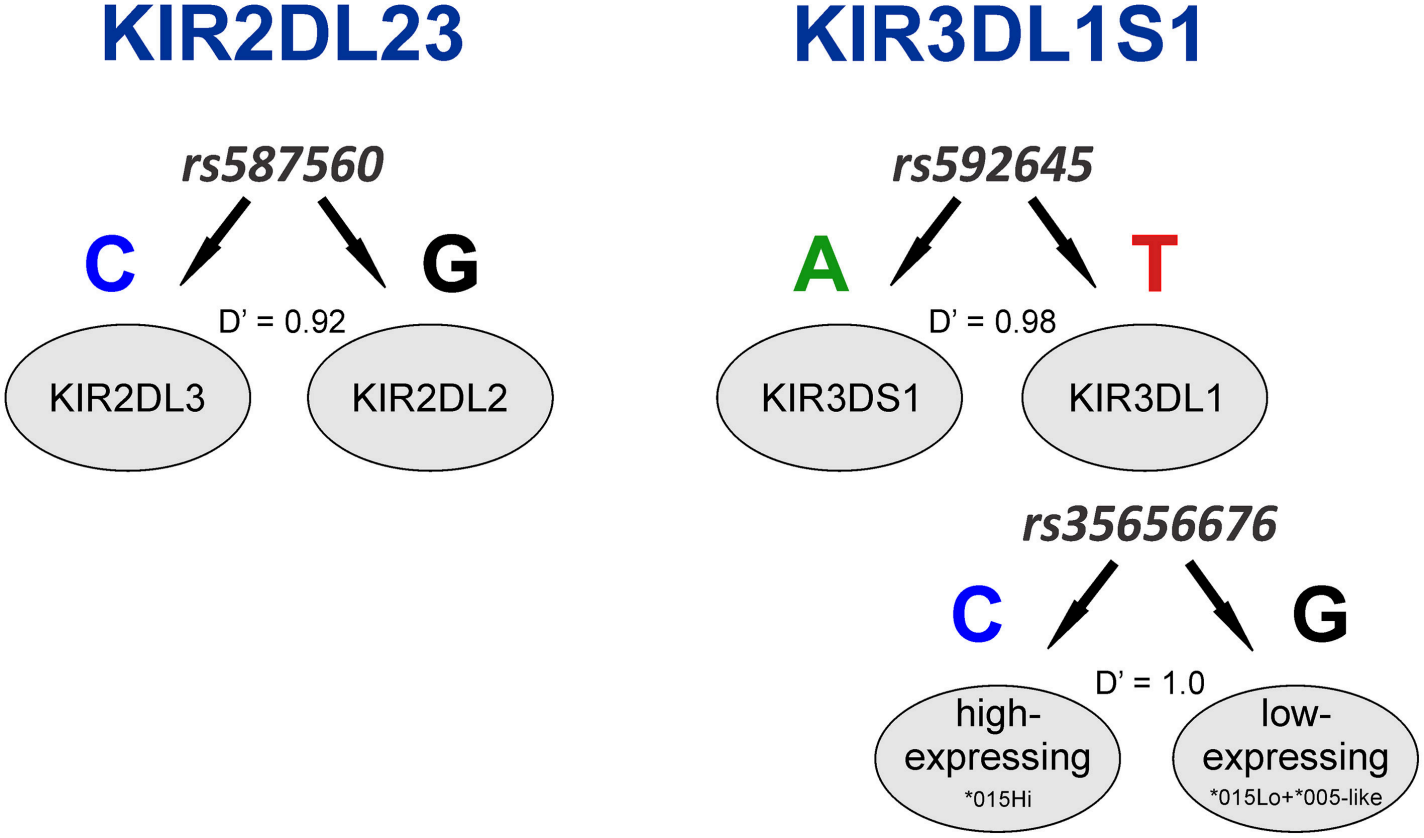
Three SNPs define *KIR2DL23* and *KIR3DL1S1* allelic lineages. The SNP *rs587560* (*KIR3DL3* intron 5) marks *KIR2DL23* allelic lineages while the pair of SNPs *rs592645* (*KIR2DL4* intron 4) and *rs35656676* (*KIR2DL4* 5' UTR) defines the major *KIR3DL1S1* lineages.

*KIR2DL2* and *KIR2DL3* had been previously treated as two separate genes but are now known to be major allelic groups of the same locus, with specific haplotypic associations. Because these allele groups have been associated with numerous diseases (41–43) as well as outcome in hematopoietic stem cell transplant (HSCT) (44), there is interest in identifying markers that distinguish them. Synthesizing the SNP data with data for *KIR* gene-content for the HGDP panel (26), we observed that the allele *rs587560C* is in strong linkage disequilibrium (LD) with (and essentially marks the presence of) *KIR2DL3* (*Cen-A* haplotypes), while the allele *rs587560G* is in linkage disequilibrium with *KIR2DL2* (*Cen-B1, Cen-B2* and *Cen-B3* haplotypes; D' = 0.92; Figure 2).

*KIR3DL1S1*, also formerly described as two genes (*KIR3DL1* and *KIR3DS1*), is possibly the most well-characterized *KIR*, with almost 200 known alleles (45) that form three major ancient lineages. The KIR3DL1^*^015 lineage is comprised of the alleles coding inhibitory receptors with the highest cell surface expression, with the exception of the low-expressing ^*^007-like subgroup. These two forms of KIR3DL1^*^015 are referred here as ^*^015Hi and ^*^015Lo. The KIR3DL1^*^005 lineage is comprised of alleles encoding low-expressing inhibitory receptors, termed ^*^005-like. Finally, the *KIR3DS1* lineage encodes activating receptors (46). We observed that the SNP *rs592645* (located in *KIR2DL4* intron 4) is a marker for the *KIR3DL1* and *KIR3DS1* allelic lineages. The variant *rs592645A* is in strong LD with the presence of *KIR3DS1* whereas the allele *rs592645T* marks *KIR3DL1* (D' = 0.98). Using high-resolution allelic genotyping for a subset of individuals (Table 1), we also analyzed the SNP data in the context of *KIR3DL1S1* alleles (Supplemental Table 1). We found that the variant *rs35656676C*, located in *KIR2DL4* 5' UTR, marks *KIR3DL1* high-expressing alleles (^*^015Hi) and *rs35656676G* marks *KIR3DL1* low-expressing alleles (^*^005-like and ^*^015Lo) (D' = 1.0; Figure 2).

### Strong Positive Selection for KIR3DL1S1 Allelic Lineages in Africa and Oceania

**Figure 3:**
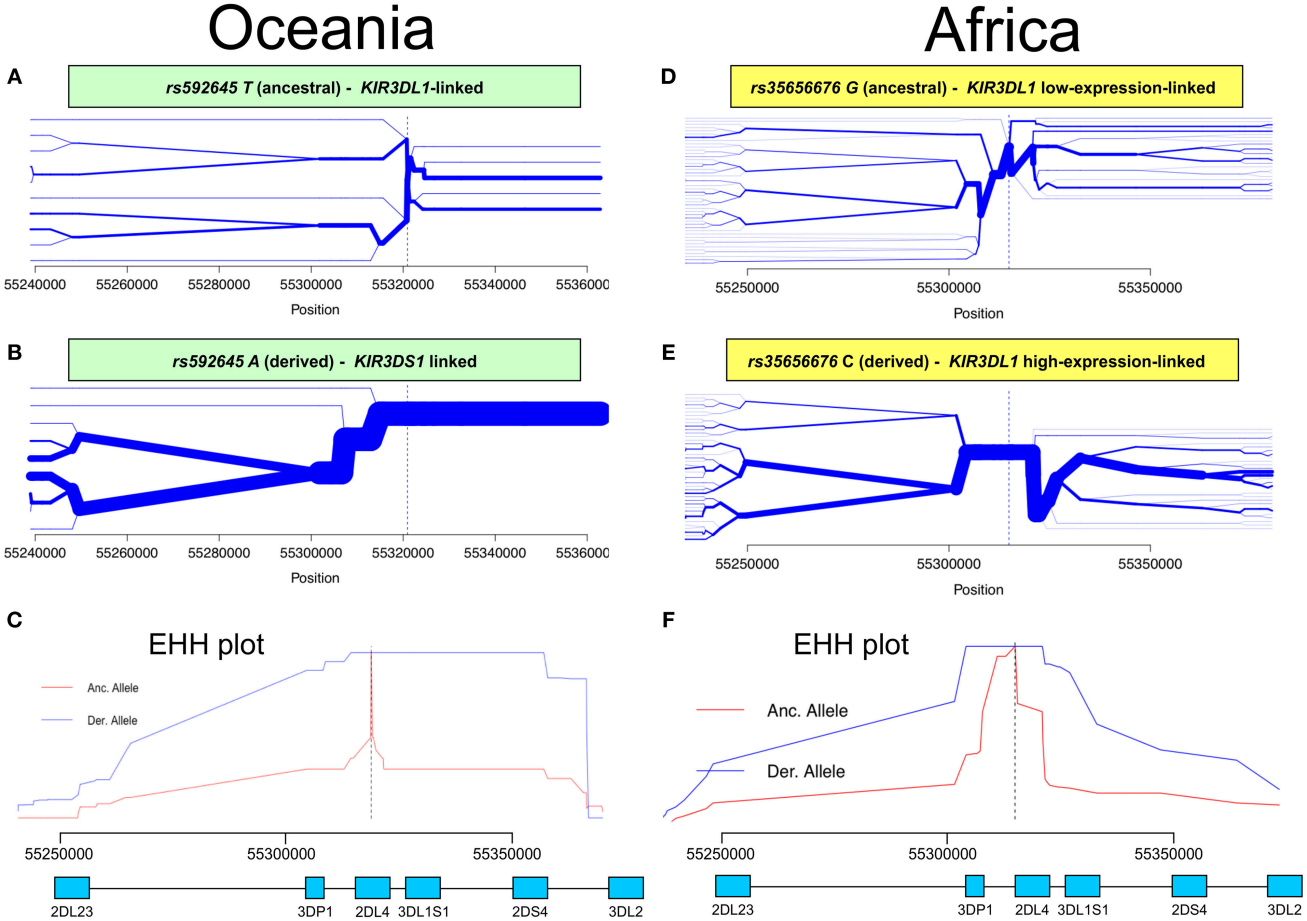
Extended haplotype homozygosity (EHH) analysis points to ongoing positive selection in Oceania and Africa. Haplotype bifurcation diagrams for ancestral **(A)** and derived **(B)** alleles of *rs592645* showing conserved haplotype associated with the derived allele, but not with the ancestral. The thickness of the line denotes haplotype frequency. **(C)**. EHH plot for *rs592645* showing decay of haplotype homozygosity in which the derived allele *A* is under selection and sweeping to fixation. Haplotype bifurcation diagrams for ancestral **(D)** and derived **(E)** alleles of *rs35656676* showing conserved haplotype associated with the derived allele, but not with the ancestral. **(F)** EHH plot for *rs35656676* showing decay of haplotype homozygosity in which the derived allele *C* is under selection and sweeping to fixation.

Having identified *KIR2DL4* SNPs as markers for *KIR3DL1S1* allelic lineages, we sought to examine them in the context of extended haplotypes to detect signatures of selection. We calculated the extended haplotype homozygosity (EHH) across nearly 500 kb flanking the *KIR* region using *rs592645* and *rs35656676* as focal SNPs. EHH detects the transmission of an extended haplotype without recombination, examining the probability of two randomly chosen chromosomes carrying a core of alleles in homozygosis (for the interval from the core region to the focal SNP) being identical by descent (36). We identified the ancient and derived alleles from the Database of Single Nucleotide Polymorphisms (dbSNP) (47) and generated bifurcation and EHH plots to visualize the range and frequency of extended haplotypes for each allele. The observed patterns point to a history of strong, recent positive selection favoring the derived alleles *rs592645* and *rs35656676* (Figure 3). Specifically, the high frequency of a conserved haplotype linked to the derived allele suggests that recent positive selection has acted to increase the frequency of the haplotype on which it originated, more rapidly than it could be broken down by recombination (36).

### Worldwide Population Frequencies of KIR3DL1 High-Expressing Alleles Correlate With Pathogen Load

**Figure 4:**
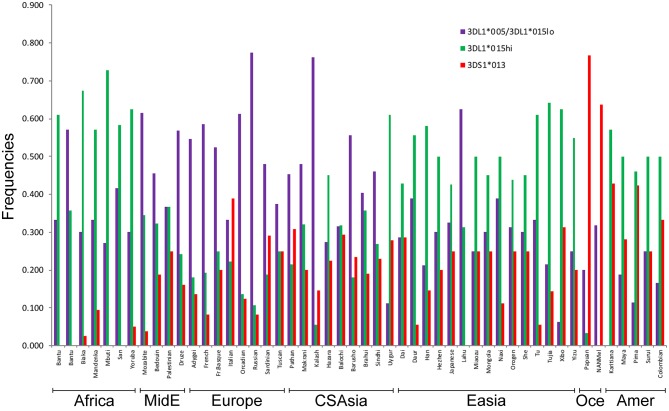
Inferred worldwide frequencies of *KIR3DL1S1* allelic lineages. The inference was based on the pair of SNPs *rs592645* and *rs35656676*, which mark the *KIR3DS1* (D' = 0.98) and *KIR3DL1* high- and low-expressing lineages (D' = 1.0), respectively. MidE, Middle East; CSAsia, Central and South Asia; EAsia, East Asia; Amer, America; Oce, Oceania.

We used the patterns of LD observed for the SNPs linked to *KIR3DL1S1* to impute the frequencies of the high- and low-expressing *KIR3DL1* lineages and *KIR3DS1* in 50 populations from the HGDP for whom SNP data were available (Figure 4). The lowest frequencies of *KIR3DS1* were observed in African populations (frequencies ranging from 0 to 0.10) and the highest in Oceanic populations (0.64 to 0.77), similar to what has been previously observed in worldwide populations (48, 49). The high-expressing *KIR3DL1* allele lineages were generally observed in higher frequencies in East Asian, Amerindian and African populations. As validation of this approach, the inferred frequencies of *KIR3DS1* in our study were compared to the previous frequencies as described in Hollenbach et al. (26), and we observed a strong correlation for these results (*r* = 0.89, *p* < 10^−7^; Supplementary Figure 1). In addition, the relatively high frequencies of *KIR3DS1* in Oceanic and Amerindian populations, as well as high frequency of low-expressing alleles in Europeans, are consistent with previous population genetics studies (3, 22, 46, 50, 51).

**Figure 5:**
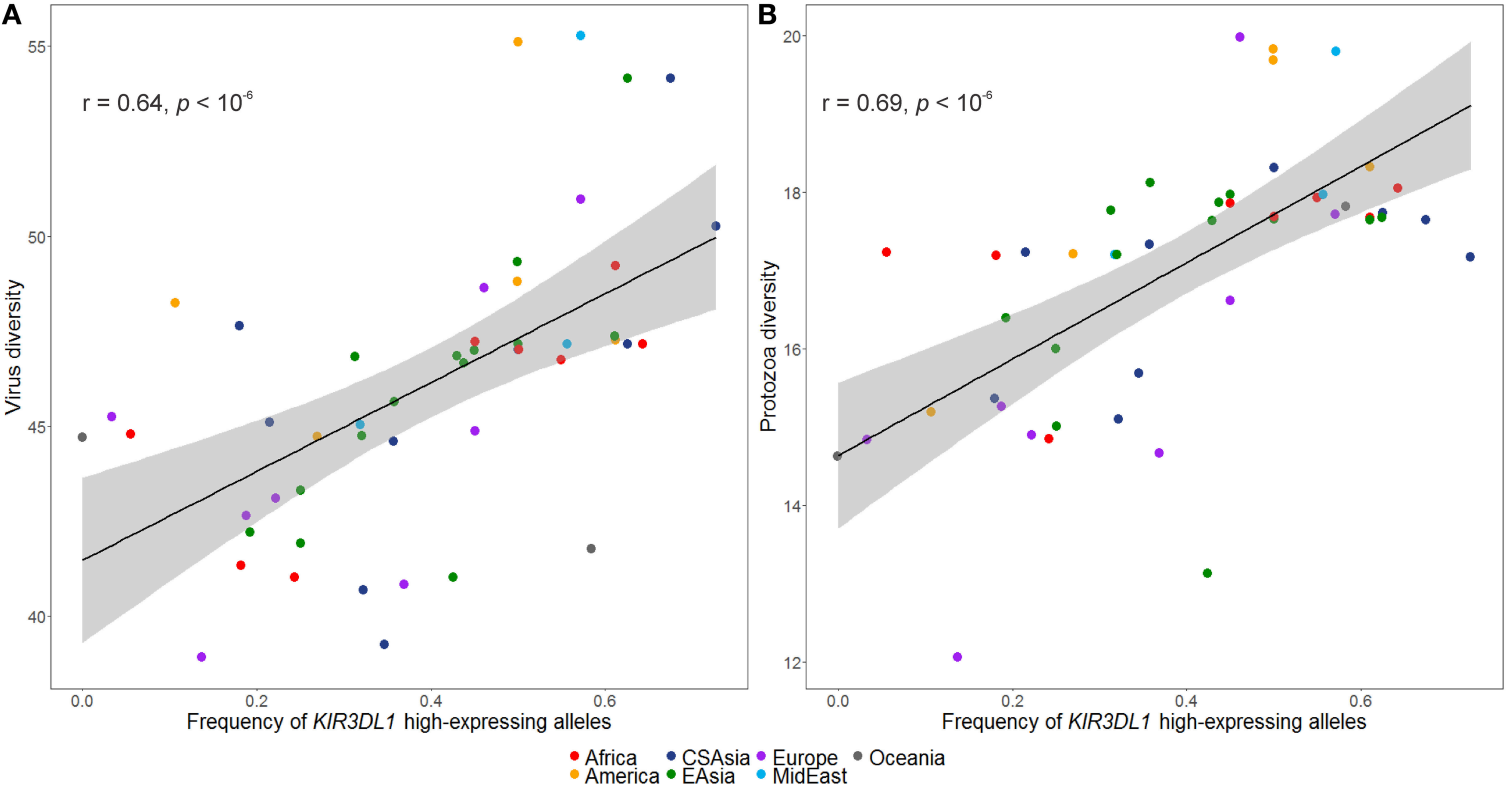
Worldwide frequencies of *KIR3DL1* high-expressing alleles correlate with virus and protozoa diversity. **(A)** Virus load correlates with frequencies of *KIR3DL1* high-expressing alleles. **(B)** Protozoa load correlates with frequencies of *KIR3DL1* high-expressing alleles. Each dot represents a population; the shaded area represents the 95% confidence interval for the regression line.

The inferred frequencies of high-expressing *KIR3DL1* alleles were then compared to previously published data quantifying pathogen diversity (given as the number of different species for a given pathogen type) in all HGDP populations (39). We found a strong and significant positive correlation between the worldwide frequencies of the high-expressing *KIR3DL1* allele lineage with virus (*r* = 0.64, *p* < 10^−6^; Figure 5A) and protozoa (*r* = 0.69, *p* < 10^−6^; Figure 5B) loads.

## Discussion

The complexity of polymorphism at the *KIR* cluster has served as a barrier to analysis in the context of whole genome diversity. Here, by leveraging publicly available data for of 650,000 SNPs in one of the most well-characterized sample collections of global populations, we present the first analysis of diversity and patterns of selection in the *KIR* region in comparison to diversity across the human genome. We merged these data with previously reported *KIR* gene-content genotyping data and derived high-resolution allelic genotyping for a subset of samples in which whole-exome or -genome sequencing data were available. We found that strong LD in the region allows exploitation of specific SNPs to mark both the presence of *KIR* haplotypes and allelic lineages of *KIR* loci and previously reported to be associated with disease and transplant outcome, as well as being under natural selection. For instance, the presence of *KIR2DL2* or *KIR2DL3* allelic lineages can be determined with 92% of accuracy by simply analyzing the SNP *rs587560* in *KIR3DL3*. By way of comparison, Vukcevic et al. (52) previously reported accuracy of 98% by imputing *KIR2DL2* and *KIR2DL3* in European populations based on several tag SNPs extracted from the Illumina Immunochip (52). It is important to note, however, that although our sample is small, our results apply to many non-European populations. Although both KIR2DL2 and KIR2DL3 molecules are inhibitory and share 96% of their amino acid sequence, it has been shown that the interaction of KIR2DL2 with HLA-C1 is stronger than the interaction with KIR2DL3 (53), and each have been variably associated with a number of diseases (42, 43). Additionally, *KIR2DL2* also marks the presence of Cen B haplotypes, which carry more activating genes than the Cen A haplotypes and have been reported to be favorable for HSCT outcome when present in donors^3^. We also showed that a pair of *KIR2DL4* SNPs, *rs592645* and *rs35656676*, define the three main *KIR3DL1S1* allelic lineages. The distinction between *KIR3DS1*, and *KIR3DL1* high- and low-expressing alleles are particularly relevant for NK cell education and disease studies, as it has been previously shown that low expression of KIR3DL1 limits the reactive potential of educated NK cells (54–56). Therefore, it is notable that these functionally relevant SNPs may serve as a proxy for these lineages in studies in which genotyping *KIR* at high-resolution is not possible.

Consistent with purifying selection, we observed lower levels of diversity in the telomeric *KIR* region in comparison to the entire genome in African and Oceanic populations relative to genome-wide diversity. In contrast, the diversity of the *KIR* centromeric region in Oceania was significantly higher in comparison to genome-wide, which could be explained by balancing selection (57). In contrast, and highlighting the considerable fluctuation of selection pressures, East Asian populations exhibit signatures of ongoing purifying selection on the centromeric region, consistent with previous work (58). Lack of signs of selection in Amerindians is in accordance with previous conclusions that demographic factors have generally a stronger effect in shaping *KIR* polymorphism in these isolated populations than natural selection (51, 59).

As this approach analyzes the variation of a specific genomic region in comparison to the whole genome in the same individuals, the differences that we observed are not likely to be explained by demographic or stochastic factors. Likewise, our results bolster previous findings that showed high diversity and balancing selection shaping the *KIR* centromeric region in African populations whereas low diversity was observed in the telomeric region in these populations(46).

We applied a robust method to detect signs of positive selection within the *KIR* telomeric region. The EHH method relies on the relationship between the frequency of an allele and the extent of linkage disequilibrium in neighboring positions. Under the neutral theory of molecular evolution, a novel variant will take a long time to reach high frequency in a population (60). Meanwhile, as a consequence of recombination, the LD between the novel variant and those in the adjacent genomic region decays significantly with time. However, in the case of strong positive selection, the frequency of an allele may increase more rapidly than the haplotype decays under recombination, resulting in large extended haplotypes at high frequencies. The patterns visualized in the bifurcation plots for *rs592645* and *rs35656676* (Figure 3) are consistent with positive selection on the derived alleles of these SNPs (36). In Oceania, there is evidence for positive selection on *rs592645A*, which marks the activating *KIR3DS1* allelic lineages. It has been suggested that high frequencies of activating *KIR* are particularly relevant for populations with an extensive history of migration (61). Specifically, the presence of *KIR3DS1* has been associated with susceptibility to several diseases (62–67), while the A haplotype (predominantly inhibitory) has been associated with protection against viral infections (68–70). The populations from the Pacific islands suffered historical mass mortality due to infectious diseases between the 16th to 19th centuries. Epidemics of smallpox, bacterial dysenteries and measles devastated the isolated populations from those islands, killing from one quarter to half of the entire population (71–77). A model proposed by Parham and Moffett (78) suggests that when populations are exposed to epidemic infections, there is a positive selection of A haplotypes. However, as the B haplotype (generally more activating than A haplotypes) are associated with successful reproduction, there is selection toward the B haplotype in subsequent generations of those who survive an epidemic. Thus, this model may explain the signature of positive selection in the Oceanic populations, which migrated to Oceania 40–50 thousand years ago, followed by episodes of epidemic infectious diseases and population expansion as well as long-term isolation from the rest of the world (79).

Our analysis was also consistent with patterns of positive selection observed in the *KIR* telomeric region in Africa. We showed evidence of positive selection toward the high-expressing *KIR3DL1* allelic lineage (marked by the allele *rs35656676C*) in African populations. These results corroborate previous work concluding that exposure to pathogens led to positive selection of more inhibitory forms of KIR3DL1 in Africans (46). Moreover, high-expressing *KIR3DL1* alleles have been associated with lower human immunodeficiency virus (HIV) viral load and slower progression to acquired immunodeficiency syndrome (AIDS) (9). In addition, expression levels of *KIR3DL1S1* play an important role in NK cell activation against HIV infected cells (54) and are critical in educating NK cells to be primed for attack (55). It is therefore tempting to speculate that extensive exposure to pathogens might be underlying positive selection for high-expressing *KIR3DL1* alleles in Africa. Likewise, malaria is a protozoan disease that has strongly impacted human evolution, particularly in Africa (80, 81), and its susceptibility has also been associated with the presence of *KIR3DL1* (82, 83). This notion of ongoing pathogen-driven positive selection in Africa is supported by the strong positive correlation that we observed between the frequencies of *KIR3DL1* high-expressing alleles with virus and protozoa loads. While the role of NK cells in viral control is well-understood, a possible mechanism underlying the correlation of *KIR* expression with protozoa load is not immediately clear; however, as with some viral infection, it could be related to immune evasion in protozoans via downregulation of HLA class I (84). At the same time, we also observed a strong correlation between virus and protozoa loads (*r* = 0.76, *p* < 10^−6^). Therefore, an alternative explanation is that selection on *KIR* is driven by viral diversity, and the association of K*IR* with protozoa load is simply a consequence of protozoan diversity tracking closely with viral diversity. Despite this limitation in providing mechanistic explanations, our results suggest that *KIR3DL1S1* pathogen-driven selection is a global phenomenon.

In conclusion, we observed geographically variable and fluctuating diversity of the centromeric and telomeric *KIR* regions among populations as well as population-specific signatures of selection. These fluctuations are likely the consequence of rapidly evolving genes that are strongly impacted by local pressures. Our findings point to the continued importance of studying *KIR* diversity and evolution across worldwide populations to improve our understanding of how this unique and complex system may contribute to human health and survival.

## Data Availability

Publicly available datasets were analyzed in this study. This data can be found here: ftp://ftp.cephb.fr/hgdp_supp15.

## Author Contributions

DA and JH drafted the manuscript. JH, PN, DA, RD analyzed the data. All authors discussed the results and contributed to the final manuscript.

### Conflict of Interest Statement

The authors declare that the research was conducted in the absence of any commercial or financial relationships that could be construed as a potential conflict of interest.

## Supplementary Material

Supplementary Figure 1 Frequencies of inferred *KIR3DS1* frequencies correlated with observed frequencies. Each dot represents a population.

Supplementary Table 1 rs35656676, rs592645 and KIR3DL1S1 genotypes.
