# Supplementary figures and images for: Fluctuating and Geographically Specific Selection Characterize Rapid Evolution of the Human KIR Region
Source: Front Immunol. 2019 May 17;10:989. doi: 10.3389/fimmu.2019.00989 (PMC6533848; doi:10.3389/fimmu.2019.00989)

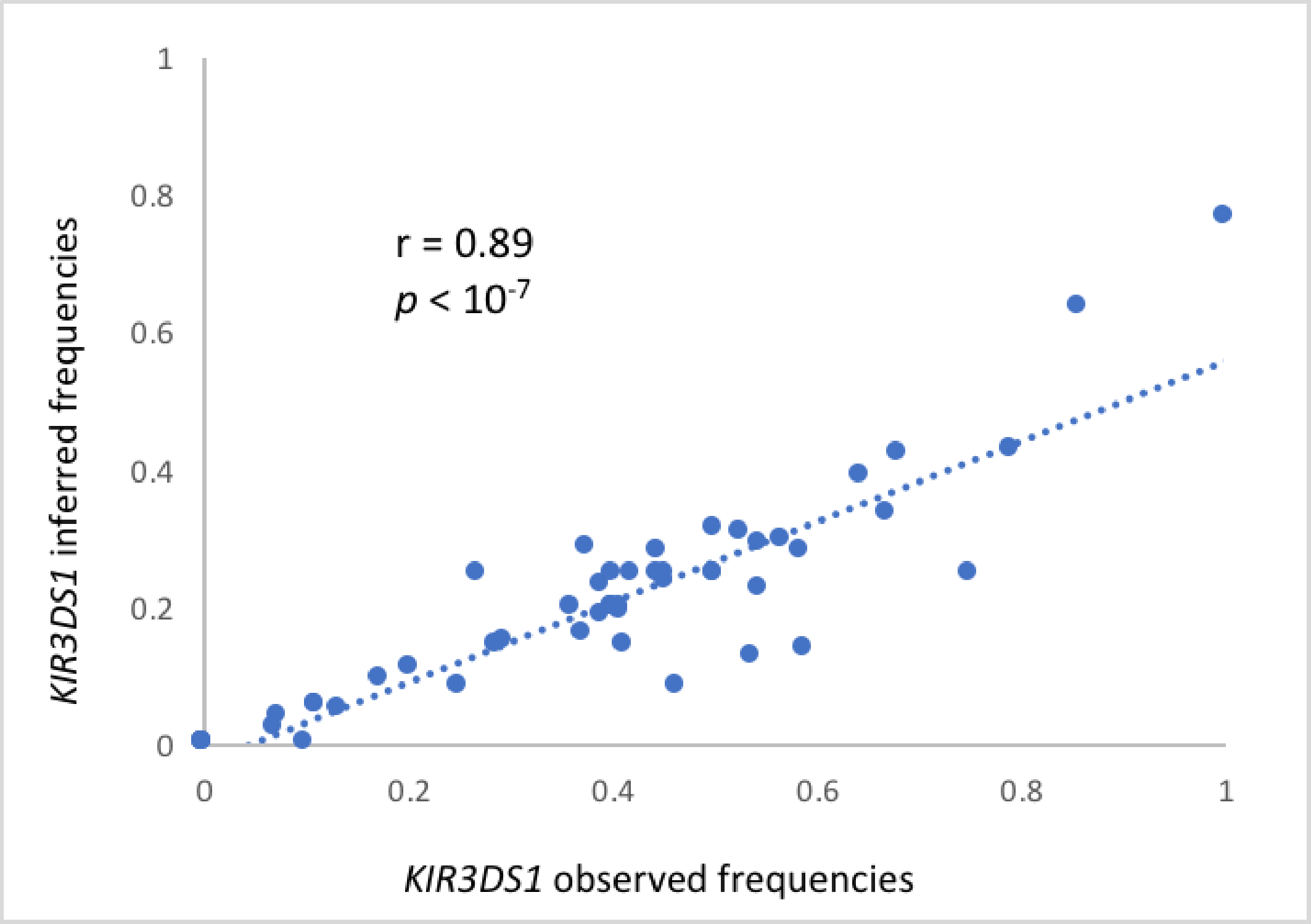

Supplement: Supplementary Figure 1 — Frequencies of inferred KIR3DS1 frequencies correlated with observed frequencies. Each dot represents a population. [file Image_1.TIF]
